# Racial difference in histologic subtype of renal cell carcinoma

**DOI:** 10.1002/cam4.110

**Published:** 2013-08-06

**Authors:** Andrew F Olshan, Tzy-Mey Kuo, Anne-Marie Meyer, Matthew E Nielsen, Mark P Purdue, W Kimryn Rathmell

**Affiliations:** 1 University of North Carolina Lineberger Comprehensive Cancer CenterChapel Hill, North Carolina; 2 Division of Cancer Epidemiology and Genetics, National Cancer InstituteBethesda, Maryland

**Keywords:** Epidemiology, histological type, incidence, race, renal cell carcinoma

## Abstract

In the United States, renal cell carcinoma (RCC) has rapidly increased in incidence for over two decades. The most common histologic subtypes of RCC, clear cell, papillary, and chromophobe have distinct genetic and clinical characteristics; however, epidemiologic features of these subtypes have not been well characterized, particularly regarding any associations between race, disease subtypes, and recent incidence trends. Using data from the Surveillance, Epidemiology, and End Results (SEER) Program, we examined differences in the age-adjusted incidence rates and trends of RCC subtypes, including analysis focusing on racial differences. Incidence rates increased over time (2001–2009) for all three subtypes. However, the proportion of white cases with clear cell histology was higher than among blacks (50% vs. 31%, respectively), whereas black cases were more likely than white cases to have papillary RCC (23% vs. 9%, respectively). Moreover, papillary RCC incidence increased more rapidly for blacks than whites (*P* < 0.01) over this period. We also observed that increased incidence of papillary histology among blacks is not limited to the smallest size strata. We observed racial differences in proportionate incidence of RCC subtypes, which appear to be increasing over time; this novel finding motivates further etiologic, clinical, molecular, and genetic studies.

Using national data, we observed a higher proportion of black renal cell carcinoma (RCC) cases with papillary histology compared to Caucasian cases. We also observed time trends in black-white incidence differences in histologic RCC subtypes, with rapid increases in the disproportionate share of black cases with papillary histology.

## Introduction

Renal cell carcinoma (RCC) comprises several distinct histological subtypes, the most commonly diagnosed including clear cell, papillary, and chromophobe. These subtypes, classically defined by histology 1, are associated with distinct molecular and genetic characteristics 2–4. Notably, clear cell RCC is associated with cytogenetic loss of chromosome 3p, encompassing four of the most commonly mutated genes in this cancer: the closely linked Von Hippel–Lindau (VHL) tumor suppressor gene which has been identified to be inactivated in up to 92% of cases 5, and the more recently recognized high-frequency mutations in PBRM1 (polybromo 1), BAP1 [BRCA1 associated protein-1 (ubiquitin carboxy-terminal hydrolase)], and SETD2 (SET domain containing 2) 6–8. In contrast, neither papillary nor chromophobe histology tumors have been associated with any of these genomic alterations. Further, the cytogenetic profile is highly distinct with papillary tumors displaying trisomy 7 and 17, and chromophobe RCC associated with multiple monosomies (characterized by collective losses of chromosomes 1, 2, 6, 10, 13, 17, and 21) 9. Not surprisingly, then, these tumors display differences in clinical manifestations, rates for recurrence, and response to targeted therapy 10–14.

Several risk factors for RCC have been consistently identified in epidemiologic studies. The better established factors include cigarette smoking, body weight, hypertension, and familial cancer syndromes 15. Cigarette smoking has been associated with a dose–response pattern of 20–30% increased risk and decrease in risk with cessation 16. Obesity has been associated with a 40% or greater elevated risk of RCC in U.S. studies 17,18. A systematic review of BMI and cancer risk found a 24% increase in the risk of RCC for every 5 kg/m^2^ increase in BMI (34% for women) 19. Hypertension has also been shown to be associated, in a dose–response manner, with an increased risk of RCC 17,20,21. Although associations have been noted, an independent effect of antihypertensive medications has not been reported 22. Other factors suggested to influence the risk of RCC include diabetes, fruit, and vegetable intake, end-stage renal disease, parity, physical activity, alcohol consumption, and trichloroethylene exposure 15. Most of these associations have been reported from studies of persons of European ancestry, although a few studies have suggested differences in the patterns of association for several risk factors, with higher risks found among blacks 23–25. A recent analysis of data from two U.S. RCC case–control studies showed that the association with obesity may vary by histologic subtype 26. However, there is limited evaluation, using national data, of the patterns of incidence of RCC subtypes, including secular trends. Such differences, if found, could reveal important subtype-specific etiological factors and identify novel targets for intervention 26,27.

Overall RCC incidence and mortality rates have previously been reported to be similar between blacks and whites, however, recent reports by Lipworth et al. 28. and Chow et al. 29. using Surveillance, Epidemiology, and End Results (SEER) Program data reported that both incidence and mortality rates were significantly higher in blacks. Moreover, incidence rates for RCC in general for African-Americans have been rising more rapidly than whites since the 1990s 29,30. A small (*n* = 204 total, 117 black) multiinstitutional study suggested that blacks had a significantly higher occurrence of papillary RCC 31, a novel observation confirmed in a recent study using SEER data, which also suggested poorer relative survival for blacks across multiple subgroups 29. A better understanding of the contribution of race to the incidence of RCC subtypes would shed light on potentially both genetic and environmental features that favor the development of these cancers. To explore RCC subtype incidence patterns over the last decade, and to examine the contribution of demographic factors, including race, on RCC subtype incidence, we examined incidence data from over 50,000 reported cases from across the U.S. over the last 9 years from the 18 registries in the SEER Program. Our analysis updates and expands a previous analysis of SEER data presented in abstract form 32.

## Material and Methods

We used data from 18 population-based registries of the SEER Program (November 2011 release) including: Alaska Native Tumor Registry, Atlanta, Connecticut, Detroit, Greater California, Greater Georgia, Hawaii, Iowa, Kentucky, Los Angeles, Louisiana, San Francisco-Oakland, San Jose-Monterey, Seattle-Puget Sound, New Jersey, New Mexico, Rural Georgia, and Utah 33.

We used the *International Classification of Diseases for Oncology, Third Edition* (ICD-O-3) site code C64.9 to identify patients with RCC diagnosed from 2001 to 2009. We focused on the three most common histologic subtypes, identified using the following ICD-O-3 histologic codes: 8310 for clear cell, 8260 for papillary, 8317 and 8270 for chromophobe. The accuracy of the subtype data entered for the SEER Program was recently examined in a cohort of 498 cases, and demonstrated a strong correlation with expert pathologic review 34. ICD-O-3 code 8312 (RCC not otherwise specified, NOS) was identified for 31,331 patients. Because of the uncertainty of the classification of these cases over time, for this report, we have excluded the RCC NOS cases from our primary analysis; however, we did perform a secondary sensitivity analysis to examine the impact of this large group of cases. Because SEER data do not capture subtype-specific classifications, such as papillary type 1, and papillary type 2, these additional levels of stratification were not examined for any of the three primary subtypes. The final cohort included a total of 52,924 patients with clear cell, papillary, and chromophobe RCC. We conducted descriptive and comparative analyses of the overall incidence among cases with the three histologic subtypes by age, sex, and race and then examined the unadjusted odds ratios of papillary and chromophobe subtypes in comparison to clear cell. We also computed age-adjusted incidence rates (cases per 100,000) standardized by Census 2000 population and tested differences in rates between the race groups, using the method of Carriere and Roos 35. This is a nonparametric method which computes a *T*^2^ statistic which follows a chi-square distribution with large sample, but does assume the data originate from a known distribution. This method can test the absolute difference between two incidence estimates from two race groups at the same time point.

We also conducted trends analysis using the Joinpoint Regression Program (version 4.0.1, NCI) 36 to examine differences in changes of incidence rates between race groups by histologic type. To do this we input the age-adjusted incidence rates in each year from 2001 to 2009 separately for whites and blacks and for each histologic type. The Joinpoint Program uses permutation tests to find a best fit of regression model with the smallest number of “joinpoints” which are distinct linear segments that differ statistically in their slopes. In addition, the program can be used to test if trends between two cohorts are statistically different (i.e., nonparallel) from each other. We obtained annual percentage change and average annual percentage changes (AAPCs) from a log-linear model in the joinpoint analysis using the logarithm of observed rates. In addition, we also performed a linear regression model using the observed rates to compute an absolute change in the rate per year by race and histologic type. Together, joinpoint analysis provided additional information on race differences based on absolute and relative changes in the incidence rate by histologic subtype. We also performed joinpoint analyses stratifying by histology and tumor size.

## Results

Among the 84,255 RCC patients, 48% of the tumors were clear cell, 37% were NOS, 10% were papillary, and 5% were chromophobe (Table 1). Excluding the NOS cases, 77% of the tumors were clear cell, 16% were papillary, and 7% were chromophobe. The proportion of RCC cases of clear cell histology among whites was higher than for blacks (50% vs. 31% respectively), whereas black cases were more likely than white cases to have papillary RCC (23% vs. 9% respectively). Whites and blacks had similar proportions of NOS cases (37% and 41%, respectively) over the study period. Compared to whites, black patients were four times as likely to have papillary RCC and twice as likely to have chromophobe RCC than clear cell RCC (Table 1). Asian or Pacific islanders were less likely to have papillary or chromophobe type than clear cell as compared with white patients (Table 1).

**Table:** Case–case comparisons of age, sex, and race distributions across renal cell carcinoma histologic subtypes.^1^

	Descriptive data by subtype					
*N* (%)					OR (95% CI)						
Clear cell (code 8310)	NOS (code 8312)	Papillary	Chromophobe	Papillary versus clear cell	Chromophobe versus clear cell
Total	40,587 (48)	31,331 (37)	8518 (10)	3819 (5)		
Age						
<45	3778 (53)	2151 (30)	670 (9)	595 (8)	1	1
45–54	8031 (53)	4733 (31)	1488 (10)	778 (5)	1.05 (0.95–1.15)	0.62 (0.55–0.69)
55–64	11,526 (52)	7371 (33)	2547 (11)	922 (4)	1.25 (1.14–1.37)	0.51 (0.46–0.57)
65–74	10,311 (49)	7762 (37)	2313 (11)	851 (4)	1.27 (1.15–1.39)	0.52 (0.47–0.59)
75+	6941 (38)	9314 (51)	1500 (8)	673 (4)	1.22 (1.10–1.35)	0.62 (0.55–0.69)
					Per 10-year increase of age (continuous variable)						
					1.05 (1.03–1.07)	0.90 (0.87–0.92)
Sex						
Male	24,902 (47)	19,351 (36)	6591 (12)	2177 (4)	1	1
Female	15,685 (50)	11,980 (38)	1927 (6)	1642 (5)	0.46 (0.44–0.49)	1.20 (1.12–1.28)
Race						
White	34,905 (50)	26,065 (37)	6168 (9)	3105 (4)	1	1
Black	2834 (31)	3796 (41)	2077 (23)	505 (5)	4.15 (3.90–4.42)	2.00 (1.81–2.22)
Asian/pacific islander	2147 (61)	1016 (29)	191 (5)	152 (4)	0.50 (0.43–0.59)	0.80 (0.67–0.94)
Other	701 (54)	454 (35)	82 (6)	57 (4)	0.66 (0.53–0.83)	0.91 (0.70–1.20)

The following ICD-O-3 codes were used to identify these subtypes: 8310 or 8312 for clear cell, 8260 for papillary, 8317 and 8270 for chromophobe. Data source is the SEER 18 registries database from November 2011 submission.

**Figure 1:**
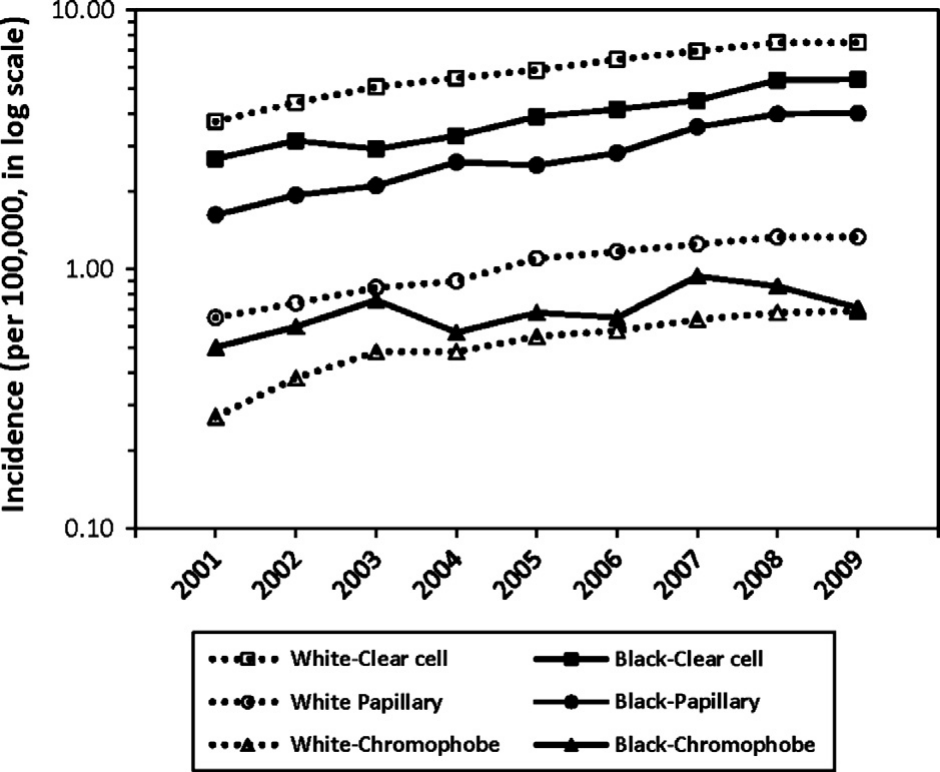
Age-adjusted renal cell carcinoma by race and histologic subtypes in 2001–2009, by black and white race. Incidence rates shown on log scale. RCC NOS (ICD-O-3 8312) cases excluded. RCC, renal cell carcinoma; NOS, not otherwise specified; ICD, international classification of diseases.

We observed an increasing trend (2001–2009) of annual age-adjusted incidence rates for all three histologic types, consistent with the increasing incidence overall, but striking differences between whites and blacks in proportionate incidence of the different subtypes (Fig. 1). For clear cell type, both groups had a twofold increase in rates from 2001 to 2009, increasing from 3.7 to 7.5 cases per 100,000 men and women for white patients and 2.7 to 5.4 for black patients.

In 2001, the beginning of the study period, blacks were roughly two times more likely to have the papillary type than whites. However, over the study period, the rise in incidence of papillary was substantially larger for black than for white patients (increasing from 1.6 to 4.0 for black patients vs. 0.7 to 1.3 for white; *P* < 0.01). By 2009, the incidence rate of papillary (4.0) approached that of clear cell (5.4) in blacks. The chromophobe subtype was more rarely diagnosed and the racial difference was no longer statistically significant in 2009.

For the trends analysis, we found that that whites and blacks had similar AAPC for clear cell, 9.6 and 9.8, respectively (data not shown). For papillary, whites had an AAPC of 9.5, smaller than the AAPC of 12.1 for blacks. These trends in subtype by race were considered parallel in the joinpoint analysis. However, the slopes based on observed incidence rates differed, with blacks having a greater increase in slope than whites (0.09; 95% CI = 0.08, 0.1 for whites and 0.30; 95% CI = 0.26, 0.34 for blacks). Moreover, among papillary tumors, blacks experienced a greater increase in larger, clinically meaningful tumors of 2–4 cm and >4 cm than among whites over the study period (0.04 vs. 0.12, *P* = 0.026; and 0.04 vs. 0.14, *P* = 0.006).

## Discussion

Using national data, we observed a higher proportion of black RCC cases with papillary histology compared to white cases, consistent with prior studies 30,32, including a prior SEER analysis 32 and an analysis of two large international case–control studies with a common central histopathologic review 26. We also observed that the well-documented rise in RCC rates in recent years reflects an increase in all of the histologies of RCC. However, we also observed dynamic black–white incidence differences in histologic RCC subtypes, and these changes in rates are not equivalent. Rather, we are observing a larger increase in the disproportionate share of black cases with papillary histology, with a widening of the gap between blacks and whites in the incidence of this subtype.

Papillary RCC is the subtype about which perhaps the least is known on the molecular level. Familial cases have been linked to mutations in the cMET proto-oncogene 37, and mutations in the fumarate hydratase gene, a key enzyme in metabolism 38. In contrast to clear cell RCC, where VHL inactivation appears to be a canonical feature in both hereditary and sporadic cases, mutations in these genes are occasionally observed, but not recognized as high-frequency events in sporadic papillary RCC. Further work by the Cancer Genome Atlas consortium and other integrated genomics efforts will shed light on the common genetic and molecular features of this tumor type. It will be essential that cases of African descent are well represented in these cohorts. The identification of commonly mutated genes or other molecular events will provide key insights into the biology of this cancer, and in particular any features differentiating white and black patients.

Established risk factors for RCC include cigarette smoking, obesity, and hypertension; some of these associations may vary by race or histologic subtype, as has been suggested in recent studies 23,26 While these risk factors have not specifically been associated with papillary RCC risk, there have not been any studies adequately powered to examine the associations separately by race and histology. Given the higher rates of tobacco exposure, obesity, and hypertension among blacks, it may be reasonable to explore the contribution that these risk factors may have on the disproportionate rise in papillary RCC in black cases. It will be important to consider genetic as well as other epidemiologic factors in determining potential mechanisms for the observed dynamic, subtype-specific incidence trends we observed. The increased use of abdominal imaging is also frequently invoked as an explanatory hypothesis for the rise RCC incidence, to the extent it leads to an increase in the incidental detection of renal masses. Changing socioeconomic status and improving access to healthcare services among blacks is an important factor to consider, but may not explain a decade long, race-specific increase in the detection of the relatively rare papillary tumor type. Our findings highlight several important population-wide features of this cancer: (1) all subtypes are on the rise, and, (2) papillary RCC, more common among black American patients, is becoming more racially disparate. Our study strengths include the use of a large dataset with standardized and systematic ascertainment and classification of RCC cancer cases. This resource permitted the estimation of nationally representative incidence rates. In addition, a standard set of demographic variables were also available. However, lifestyle, environmental, and medical conditions are not included in the SEER data and were not available for this analysis.

The clinical significance of the observed larger and disproportionately rising share of papillary cases among blacks warrants further consideration, especially given the evidence that papillary and chromophobe RCC are associated with a better prognosis than clear cell RCC. Because of evidence that most of the increases in RCC incidence in recent decades have been in small (<4 cm, stage T1a) and very small (<2 cm) localized renal masses, we examined the size distribution of papillary cases, by race. Among T1a cases, the proportion of <2 cm and 2–4 cm masses was higher among whites versus blacks (14.4% vs. 11.9% and 38.8% vs. 34.2%, respectively), whereas the proportion of masses >4 cm was higher in blacks (46/8% vs. 53.8%, respectively). Furthermore, the slope of increased incidence in these groups in the joinpoint analysis was not significantly different between the groups in the <2 cm stratum, whereas the slope of increased incidence of 2–4 cm and >4 cm tumors was significantly higher among blacks over the study period (0.04 vs. 0.12 [*P* = 0.026] and 0.04 vs. 0.14 [*P* = 0.006], respectively). These data suggest that the observed increased incidence of papillary histology among blacks is not limited to the smallest, potentially clinically insignificant size strata.

Additionally, although a standard pathologic review protocol is employed across the study areas some uncertainty in histologic classification remains. A large proportion of cases were classified as ICD-O-3 code 8312 (RCC NOS). In our original dataset code 8312 comprised 31,331 (37%) of RCC patients. A recent study of the SEER-assigned histology compared to classification by an independent pathologist found that the majority of cases with the SEER-assigned ICD-O-3 code 8312 (RCC, NOS) were classifiable as clear cell 34. We conducted a sensitivity analysis by comparing the age-adjusted rate of the clear cell subtype using code 8312 alone and then combining 8312 with clear cell (8310) (data not shown). The results for our sensitivity analysis of the combined (ICD-O-3 8310 and 8312) group are generally similar to those for 8312 alone, including an elevated odds ratio for papillary among blacks (data not shown). It appears that black patients are also more likely to be diagnosed with ambiguous histopathologic codes. Further investigation of these classification practices that may impact classification among this subtype is warranted.

Novel targeted therapeutics are rapidly being generated for RCC, including new treatments being tested for use in subtype-specific scenarios, such as MET inhibitors for use in papillary RCC. These findings highlight the growing need to develop targeted therapies in order to address issues of disparity in health care. More specifically, it demonstrates the importance of developing better methods of detection or prevention of papillary RCC among black Americans to be developed, and for improved methods of subtype determination to be put forward, potentially including molecular classification schemes, to ensure that patients receive optimal care. Future epidemiological, clinical, and genetic studies are needed to advance the understanding of RCC subtypes such that a clearer appreciation of the tumor subtype profile may provide new insights into prevention, screening, or therapy.

## Conflict of Interest

None declared.
